# Revolutionizing Maternal Health: The Role of Artificial Intelligence in Enhancing Care and Accessibility

**DOI:** 10.7759/cureus.69555

**Published:** 2024-09-16

**Authors:** Smruti A Mapari, Deepti Shrivastava, Apoorva Dave, Gautam N Bedi, Aman Gupta, Pratiksha Sachani, Paschyanti R Kasat, Utkarsh Pradeep

**Affiliations:** 1 Obstetrics and Gynecology, Jawaharlal Nehru Medical College, Datta Meghe Institute of Higher Education & Research, Wardha, IND; 2 Medicine, Jawaharlal Nehru Medical College, Datta Meghe Institute of Higher Education & Research, Wardha, IND; 3 Radiodiagnosis, Jawaharlal Nehru Medical College, Datta Meghe Institute of Higher Education & Research, Wardha, IND

**Keywords:** artificial intelligence, healthcare accessibility, maternal health, predictive analytics, pregnancy complications, telemedicine

## Abstract

Maternal health remains a critical global health challenge, with disparities in access to care and quality of services contributing to high maternal mortality and morbidity rates. Artificial intelligence (AI) has emerged as a promising tool for addressing these challenges by enhancing diagnostic accuracy, improving patient monitoring, and expanding access to care. This review explores the transformative role of AI in maternal healthcare, focusing on its applications in the early detection of pregnancy complications, personalized care, and remote monitoring through AI-driven technologies. AI tools such as predictive analytics and machine learning can help identify at-risk pregnancies and guide timely interventions, reducing preventable maternal and neonatal complications. Additionally, AI-enabled telemedicine and virtual assistants are bridging healthcare gaps, particularly in underserved and rural areas, improving accessibility for women who might otherwise face barriers to quality maternal care. Despite the potential benefits, challenges such as data privacy, algorithmic bias, and the need for human oversight must be carefully addressed. The review also discusses future research directions, including expanding AI applications in maternal health globally and the need for ethical frameworks to guide its integration. AI holds the potential to revolutionize maternal healthcare by enhancing both care quality and accessibility, offering a pathway to safer, more equitable maternal outcomes.

## Introduction and background

Maternal health is a critical component of public health, focusing on the well-being of women during pregnancy, childbirth, and the postpartum period. Ensuring optimal maternal health benefits the mother and has far-reaching implications for the child, family, and broader society [1]. Healthy pregnancies reduce the risk of complications for both the mother and infant, leading to better long-term health outcomes. Furthermore, maternal health is a key indicator of the overall effectiveness of healthcare systems, as it encompasses aspects like access to care, quality of services, and the availability of timely interventions [2]. Despite advancements in medicine, many women globally still face significant risks during pregnancy and childbirth, making maternal health a priority in healthcare policies and innovations [3]. Despite global efforts to improve maternal health, many countries face substantial challenges. Each year, approximately 295,000 women die from complications related to pregnancy and childbirth, with the vast majority of these deaths occurring in low- and middle-income countries [4]. Factors such as inadequate healthcare infrastructure, lack of trained healthcare providers, and socioeconomic barriers contribute to these outcomes. In rural and underserved areas, healthcare access is often limited, leading to delays in diagnosis, insufficient prenatal care, and a lack of emergency obstetric services [5].

In higher-income countries, disparities in maternal healthcare still exist, particularly among marginalized populations. Racial, ethnic, and socioeconomic inequalities persist, leading to higher maternal mortality rates among certain groups. For example, in the United States, Black women are disproportionately affected, with maternal mortality rates three to four times higher than those of white women. These challenges emphasize the need for innovative solutions that can address gaps in care and ensure that all women receive equitable, high-quality maternal healthcare [6]. Artificial intelligence (AI) has emerged as a transformative technology in various industries, and healthcare is no exception. AI encompasses a range of technologies, including machine learning, natural language processing, and predictive analytics, which can process vast amounts of data to identify patterns and support decision-making [7]. In healthcare, AI applications have demonstrated the potential to improve diagnostic accuracy, personalize treatment plans, and enhance patient monitoring. AI’s ability to analyze complex medical data and its capacity to continuously learn and improve from new information have made it a promising tool for addressing some of the most pressing challenges in maternal health. From predicting pregnancy complications to facilitating remote monitoring and care, AI has the potential to revolutionize maternal healthcare by making it more proactive, personalized, and accessible [7].

This review aims to examine how AI can enhance maternal healthcare and increase accessibility to quality care for women worldwide. By analyzing AI-driven innovations, this review will explore how these technologies can improve early detection of complications, provide personalized care, and bridge gaps in healthcare access. Additionally, this review will consider the ethical challenges and limitations associated with AI in maternal healthcare, as well as future directions for research and implementation. Through this exploration, the potential of AI to revolutionize maternal health and address existing disparities will be evaluated, offering insights into how these technologies can shape the future of care for mothers and their children.

## Review

The current state of maternal healthcare

Maternal mortality remains a pressing global health issue, with significant disparities across various regions [8]. The World Health Organization (WHO) reported that approximately 287,000 women died during or shortly after pregnancy and childbirth in 2020, with nearly 95% of these deaths occurring in low- and middle-income countries [8]. From 2000 to 2020, the global maternal mortality ratio (MMR) declined by approximately 34%, from 339 to 223 deaths per 100,000 live births. However, the reduction rate has slowed recently, and some regions have even experienced an increased MMR. In the United States, the maternal mortality rate was 32.9 deaths per 100,000 live births in 2021, significantly higher than in other high-income countries, indicating systemic issues within the healthcare system [9]. Racial disparities are also stark, with Black women being about three times more likely to die from pregnancy-related causes compared to their white counterparts [10]. Geographic, socioeconomic, and racial factors influence maternal healthcare disparities. The WHO reports that sub-Saharan Africa accounted for approximately 70% of global maternal deaths in 2020, with MMRs in low-income countries reaching 430 per 100,000 live births compared to just 13 in high-income countries. Socioeconomic status plays a crucial role, as women in impoverished communities often lack access to quality healthcare services [11]. Racial disparities persist within countries as well, particularly in the U.S., where systemic racism contributes to higher mortality rates among Black and Indigenous women. These disparities underscore the urgent need for targeted interventions to ensure equitable access to maternal healthcare for all women [12]. Several challenges impede the provision of timely and high-quality maternal care. Access to care remains a significant barrier, especially for women in rural and underserved areas who often struggle to obtain prenatal and postnatal services. The quality of care can also be compromised by inadequate training of healthcare providers and a lack of resources, leading to poor maternal outcomes [13]. Health system inequities further exacerbate these challenges, as marginalized groups frequently receive substandard care. Societal barriers, including cultural beliefs and practices, can also hinder women's access to necessary maternal health services. The COVID-19 pandemic has added complexity by disrupting maternal healthcare services and increasing risks for pregnant women [14]. Technology has become a crucial tool in addressing these challenges in maternal healthcare. Telehealth services have gained prominence, particularly during the pandemic, enabling women in remote areas to access prenatal and postnatal care without extensive travel. Digital health platforms offer educational resources, monitor maternal health indicators, and facilitate communication between patients and healthcare providers, ensuring timely interventions [15]. Moreover, AI and data analytics are used to identify at-risk pregnancies and enhance care delivery through predictive modeling and personalized healthcare plans. Community-based programs, such as the Black Maternal Health Momnibus Act, aim to leverage technology to improve data collection and maternal health services, particularly for women of color [15].

AI-driven innovations in maternal health

AI revolutionizes maternal healthcare by improving early detection, monitoring, supporting clinical decision-making, and reducing diagnostic errors. One of the most notable advancements is AI's capacity to predict complications such as preeclampsia, gestational diabetes, and preterm labor [16]. By analyzing large datasets, AI algorithms can identify patterns and risk factors indicative of potential complications, enabling healthcare providers to intervene early. Additionally, AI-powered risk stratification tools categorize expectant mothers according to their risk levels, allowing for the development of personalized care plans tailored to individual needs [17]. In prenatal and postnatal monitoring, AI-driven wearable devices and remote monitoring technologies facilitate continuous tracking of maternal and fetal health parameters. These devices collect real-time data, which AI-based platforms analyze to issue alerts for deviations from normal health indicators. This technology improves the quality of care and extends maternal healthcare services to remote and underserved areas, ensuring that expectant mothers receive timely support regardless of their location [18]. AI also plays a vital role in healthcare providers' clinical decision-support systems. By enhancing clinical decision-making during pregnancy, labor, and postpartum care, AI tools assist clinicians with informed choices based on comprehensive data analysis. AI can create personalized care plans for at-risk pregnancies that address the specific health profiles of mothers and their babies, ultimately improving outcomes and reducing the risk of complications [19]. Moreover, AI is transforming the reduction of diagnostic errors through advanced image analysis in ultrasound and MRI. AI algorithms can accurately analyze medical images, detecting abnormalities the human eye may overlook. This capability aids in the early detection of fetal complications and supports healthcare providers in making more informed decisions, leading to improved patient outcomes [20]. Despite the immense potential of AI in maternal healthcare, several challenges persist. Data quality, ethical considerations, regulatory complexities, and potential algorithmic bias must be addressed to ensure AI's safe and effective integration into clinical practice. Building trust among healthcare professionals and expectant mothers is crucial for successfully implementing these technologies [21]. The future of AI in maternal healthcare is promising, with advancements expected in personalized and adaptive healthcare solutions [16]. Integrating AI with wearable devices and home monitoring solutions is anticipated to enhance the precision and scope of fetal anomaly detection, leading to better health outcomes for mothers and their babies. As technology evolves, it is essential to fully leverage its potential while addressing challenges to ensure that all women receive the quality care they deserve during pregnancy and beyond [16]. AI-driven innovations transforming maternal health and applications and impact are detailed in Table 1.

**Table 1:** AI-driven innovations transforming maternal health: applications and impact Source: [18,22-26]

AI Innovation	Application in Maternal Health	Impact/Benefits	Example/Case Study
Predictive Analytics for Complications	Early prediction of preeclampsia, gestational diabetes, and preterm labor	Early intervention, improved maternal and fetal outcomes	AI models predicting preeclampsia based on EHR data
AI-Powered Ultrasound Imaging	Automated analysis of fetal images for detecting anomalies	Increased accuracy and reduced need for specialized radiologists	AI-based tools like Qure.ai for fetal growth monitoring
Telemedicine and AI Chatbots	Virtual prenatal consultations and 24/7 AI-based support	Improved access to care, especially in rural/underserved areas	AI chatbots like Ada Health providing maternal care support
Wearable Sensors and AI Integration	Monitoring maternal vitals and fetal movements	Real-time health data tracking, personalized care recommendations	AI-integrated wearables for continuous monitoring of vitals
AI in Genomic Analysis	Screening for genetic conditions in the fetus	Early detection of potential genetic disorders, better planning	AI-driven genomic analysis platforms for prenatal screening
AI-Enhanced Postpartum Care	Mental health monitoring and detection of postpartum depression	Timely intervention reduced maternal mental health issues	AI algorithms identifying early signs of postpartum depression

Improving maternal care accessibility through AI

AI is increasingly recognized as a transformative force in enhancing accessibility to maternal care, particularly in low-resource settings. By leveraging AI technologies, healthcare systems can address significant gaps in maternal health services, thereby improving the quality and reach of care. This is especially critical in rural and underserved communities where access to healthcare is often limited [27]. One of AI's most promising applications is telemedicine and remote care. AI-powered telehealth solutions can bridge gaps in healthcare access by enabling pregnant women in remote areas to receive timely consultations and monitoring without the need for long-distance travel [28]. These technologies allow healthcare providers to offer specialized care remotely, ensuring that expectant mothers can access essential services even without local facilities. Additionally, AI-driven virtual assistants can offer personalized support by answering health-related questions and sending reminders for appointments and medication, thus empowering women to manage their health more effectively [28]. AI also enhances health literacy and patient education. Chatbots and mobile applications designed to deliver maternal health information provide 24/7 access to critical resources [24]. These tools are invaluable for educating expectant mothers about prenatal care, warning signs, and healthy practices during pregnancy. Furthermore, natural language processing (NLP) enables these systems to offer tailored health advice based on individual circumstances, enhancing the personalization of care and ensuring that women receive relevant information that meets their specific needs [24]. Moreover, AI is crucial in optimizing resource allocation and workforce management in healthcare settings. In low-resource environments, where healthcare workers are often in short supply, AI can help streamline operations by predicting patient needs and optimizing service delivery [29]. For instance, AI algorithms can analyze data to identify areas with high demand for maternal health services, facilitating more effective planning and deployment of resources. Additionally, AI can assist in managing healthcare logistics, ensuring that essential supplies and personnel are allocated efficiently to meet the needs of pregnant women [29]. Despite the potential benefits, integrating AI into maternal healthcare presents several challenges. Data quality, ethical considerations, and the need for explainable AI solutions must be addressed to ensure the effective adoption of these technologies. Building trust among healthcare professionals and patients is essential for successfully implementing AI-driven solutions [30]. As AI continues to evolve, it promises to significantly improve maternal health outcomes by facilitating early detection of complications, enhancing patient engagement, and ultimately saving lives in vulnerable populations [30]. Enhancements in maternal care accessibility through AI-driven solutions are detailed in Table 2.

**Table 2:** Enhancing maternal care accessibility through ai-driven solutions Source: [30-35]

AI Solution	Application	Accessibility Benefit	Example/Case Study
AI-Powered Telehealth Platforms	Remote consultations for prenatal and postnatal care	Provides access to care in remote and underserved areas	Babylon Health and telemedicine platforms for maternal care
AI-Based Virtual Assistants	24/7 access to information and symptom checkers	Reduces dependency on in-person consultations for basic queries	AI chatbots like Florence offer maternal health guidance
Mobile Health (mHealth) Apps with AI	Maternal health tracking, reminders for appointments, and medication	Enables continuous care management for women in low-resource areas	mHealth apps integrated with AI for pregnancy monitoring
AI-Driven Diagnostics in Low-Resource Settings	Portable diagnostic tools for prenatal screenings	Increases access to early diagnosis where specialized facilities are lacking	Portable AI ultrasound devices used in rural clinics
AI-Based Decision Support Systems	Assisting healthcare workers in making informed decisions	Empower frontline workers in areas with limited expertise	AI decision-support tools aiding midwives in maternal care
AI for Language Translation	Real-time translation of healthcare information and consultations	Overcomes language barriers in multicultural or migrant communities	AI translation services for diverse patient populations

AI for addressing maternal mental health

AI is making significant advancements in the detection of perinatal mental health disorders, including postpartum depression and anxiety. AI can use machine learning algorithms to analyze data from various sources, such as electronic health records, patient-reported outcomes, and social media activity [36]. This analysis aids in identifying women at risk of developing postpartum mental health issues, allowing for early intervention by healthcare providers. Early detection is crucial as it enables the creation of personalized care plans tailored to each mother’s specific needs, ultimately improving outcomes for both mothers and their infants [37]. Beyond detection, AI-based digital platforms are being developed to facilitate intervention for early maternal mental health concerns. These platforms often feature AI chatbots and virtual assistants that engage with expectant and new mothers, screen for symptoms of mental health conditions, and provide customized resources and support [38]. By making mental health support more accessible and convenient, these AI-powered tools aim to reduce barriers to care, ensuring that mothers receive timely assistance. This proactive approach can significantly enhance maternal well-being during the perinatal period [38]. AI is also revolutionizing the provision of psychological support for mothers. Virtual support groups and AI-driven mental health resources are emerging, offering 24/7 access to information, coping strategies, and peer support [39]. These platforms create safe, non-judgmental spaces for mothers to share their experiences and connect with others who understand the challenges of parenthood. Such a sense of community can be invaluable for new mothers, helping alleviate feelings of isolation and anxiety that often accompany motherhood [39]. Moreover, AI-enabled therapy bots are being developed to provide on-demand mental health support. These chatbots, trained on extensive datasets of therapeutic conversations, can engage in natural language interactions to offer emotional support and cognitive-behavioral therapy techniques [40]. While these AI-driven tools are not intended to replace human therapists, they can help bridge the gap in access to mental health care, especially for mothers in underserved areas. AI-enabled therapy bots can empower mothers to seek further professional help when necessary [40]. The role of AI in addressing maternal mental health challenges is detailed in Table 3.

**Table 3:** Leveraging AI to address maternal mental health challenges Source: [41-46]

AI Solution	Application in Maternal Mental Health	Impact/Benefits	Example/Case Study
AI-Powered Mental Health Screening Tools	Early detection of postpartum depression and anxiety disorders	Enables early intervention and personalized mental health support	AI-based tools like Woebot for detecting postpartum depression
Chatbots for Mental Health Support	24/7 access to emotional support and mental health resources	Provides real-time, anonymous support for new mothers experiencing stress	AI-based chatbots like Wysa for maternal mental health support
AI-Driven Predictive Models	Identifying risk factors for maternal mental health conditions	Helps healthcare providers intervene early to prevent severe conditions	Predictive models analyzing risk based on social and clinical data
Wearable Devices with AI Integration	Monitoring stress, sleep patterns, and emotional health of new mothers	Offers continuous monitoring and timely alerts for mental health risks	AI-enabled wearables tracking mental health indicators
Virtual Cognitive Behavioral Therapy (CBT)	Delivering AI-guided CBT for managing postpartum depression	Provides accessible, cost-effective therapy options for new mothers	AI platforms delivering virtual CBT for postpartum depression
Natural Language Processing (NLP) in Counseling	Analyzing speech and text for emotional distress during consultations	Enhances the ability to detect mental health issues during routine checkups	NLP tools identifying emotional distress in verbal communication

Ethical considerations and challenges of AI in maternal health

While AI has the potential to revolutionize maternal healthcare, it also presents significant ethical considerations that must be addressed to ensure its responsible use. One primary concern is data privacy and security. Maternal healthcare involves managing sensitive information such as medical records, genetic data, and personal details [47]. Protecting this information is crucial, as breaches could have serious consequences, including identity theft and discrimination. Robust data privacy and security measures are essential to safeguard the rights of expectant mothers [48]. Compliance with the Health Insurance Portability and Accountability Act (HIPAA) in the United States and the General Data Protection Regulation (GDPR) in the European Union is critical when developing and deploying AI systems in maternal healthcare. Adhering to these regulations protects patient data and builds trust in AI technologies [48]. Another critical ethical consideration is bias and fairness in AI algorithms. AI models can inadvertently perpetuate existing biases related to race, ethnicity, and socioeconomic status, potentially leading to inequitable maternal health outcomes. For example, if the data used to train AI systems predominantly represents certain demographic groups, the resulting algorithms may not accurately reflect or address the needs of underrepresented populations [49]. AI developers need to include diverse groups of women in the development process to promote fairness and prevent unintended biases. This approach will help ensure that AI-driven solutions contribute to equitable maternal health outcomes for all expectant mothers, regardless of their background [50]. The role of human oversight in AI-based care is another significant ethical consideration. While AI can enhance decision-making, it is important not to rely overly on these technologies in critical situations. For instance, decisions regarding diagnosis and treatment in maternal healthcare should not depend solely on AI recommendations. Maintaining the human element in care is essential, as healthcare providers bring invaluable experience, empathy, and intuition to patient interactions. Balancing AI capabilities with clinician expertise is vital to ensure that AI systems complement and enhance the work of healthcare providers rather than replace them entirely. This collaborative approach can improve health outcomes while preserving the essential human touch in maternal care [51]. Ethical considerations and challenges in implementing AI in maternal health are detailed in Table 4.

**Table 4:** Ethical considerations and challenges in implementing AI in maternal health Source: [52-59]

Ethical Consideration/Challenge	Description	Potential Impact on Maternal Health	Possible Solutions/Approaches
Data Privacy and Security	Protecting sensitive health information of mothers and infants	Risk of data breaches, loss of trust in AI systems	Implementing robust data encryption, strict access controls
Bias in AI Algorithms	AI models trained on biased data may lead to disparities in maternal care	Unequal access to care, worsening health outcomes for marginalized groups	Using diverse and representative data sets for training AI models
Informed Consent and Autonomy	Ensuring mothers understand how AI is used in their care	Lack of understanding may lead to misinformed decision-making	Clear communication, ensuring transparent AI application in care
Accountability for AI Decisions	Determining who is responsible for errors in AI-driven care	Ambiguity in liability for AI misdiagnoses or incorrect recommendations	Establishing clear guidelines and legal frameworks for AI accountability
Access to AI Technology	Ensuring equitable access to AI-driven maternal health innovations	The digital divide may leave low-resource communities behind	Government and NGO initiatives to provide AI technology to underserved regions
Reliability and Accuracy of AI Tools	Ensuring AI systems produce consistent and accurate results across diverse populations	Unreliable AI predictions may lead to harmful health outcomes	Rigorous validation and testing of AI tools across different demographics
Ethical Use of Predictive Analytics	Using AI to predict complications in a way that respects patient dignity	Predictive analytics could lead to over-medicalization or anxiety	Ethical frameworks guiding AI use in sensitive predictions
Human Oversight in AI-Driven Care	Balancing AI recommendations with healthcare professionals' expertise	Over-reliance on AI could reduce personalized care	Ensuring human oversight and final decision-making in AI-supported care

Future directions and research opportunities

Integrating AI with wearable technology and the Internet of Things (IoT) represents a promising frontier in maternal healthcare. AI-powered wearables can continuously monitor maternal and fetal health parameters, such as heart rate, blood pressure, and fetal movement, offering real-time insights and alerts to healthcare providers and expectant mothers [60]. This ongoing monitoring can alleviate the burden on healthcare facilities, especially in remote and underserved areas where access to quality prenatal care is limited. By combining AI algorithms with smart IoT devices, comprehensive maternal health monitoring systems can be developed, allowing for proactive interventions and personalized care tailored to each mother’s specific health profile [61]. Expanding AI-driven solutions will be crucial as the global community works towards achieving the Sustainable Development Goals (SDGs) related to maternal and child health. AI algorithms can identify high-risk pregnancies, predict complications, and guide timely interventions, particularly in low-resource settings [62]. Integrating AI into global maternal health programs can significantly enhance their reach and impact, leading to improved health outcomes for mothers and their babies worldwide. This expansion will require collaboration among governments, NGOs, and technology developers to ensure that AI solutions are accessible and effective across diverse cultural and socioeconomic contexts [63].

AI's capacity to analyze vast datasets and identify complex patterns positions it as a powerful tool for predicting long-term maternal and child health outcomes. By employing machine learning techniques, AI systems can reveal intricate relationships between factors such as maternal health history, lifestyle choices, and environmental influences and their impact on future well-being [64]. This predictive ability can inform personalized care plans and interventions, enabling healthcare providers to address potential health issues proactively and improve overall health trajectories for mothers and their children. Understanding these long-term outcomes can also help shape public health policies and optimize resource allocation to support maternal and child health [65]. As AI-driven solutions evolve in maternal healthcare, conducting rigorous clinical trials and validation studies is essential to ensure their safety, efficacy, and practical utility. Researchers and healthcare providers must work together to design and implement well-structured studies that evaluate the performance of AI algorithms in real-world settings [66]. This process will foster trust and confidence among healthcare professionals and expectant mothers, facilitating the adoption of AI technologies in maternal care. By validating these technologies through clinical trials, the maternal healthcare community can establish evidence-based practices that enhance care quality and accessibility, ultimately leading to improved health outcomes for mothers and their infants [67].

## Conclusions

In conclusion, integrating AI into maternal healthcare holds immense potential to transform the quality and accessibility of care for women worldwide. By leveraging AI’s capabilities in early detection, personalized treatment, and remote monitoring, maternal health outcomes can be significantly improved, reducing complications and mortality rates. AI-driven innovations, such as predictive models and telemedicine platforms, offer solutions to address the longstanding challenges of unequal access to care, particularly in underserved regions. However, to fully realize AI's benefits, addressing ethical concerns, such as data privacy, algorithmic bias, and the need for human oversight in critical decision-making, is crucial. As advancements in AI continue to unfold, responsible implementation and further research will be key to ensuring that these technologies are used effectively and equitably. Ultimately, AI has the potential to revolutionize maternal health, fostering a future where every woman receives timely, high-quality care regardless of her location or background.
